# Tuberculosis treatment within differentiated service delivery models in global HIV/TB programming

**DOI:** 10.1002/jia2.25809

**Published:** 2021-10-28

**Authors:** Cuc H. Tran, Brittany K. Moore, Ishani Pathmanathan, Patrick Lungu, N. Sarita Shah, Ikwo Oboho, Teeb Al‐Samarrai, Susan A. Maloney, Anand Date, Andrew T. Boyd

**Affiliations:** ^1^ Division ofHIV & Global Tuberculosis U.S. Centers for Disease Control and Prevention Atlanta Georgia USA; ^2^ National TB and Leprosy Programme Ministry of Health Lusaka Zambia; ^3^ Department of Global Health Emory University Rollins School of Public Health Atlanta Georgia USA; ^4^ Office of the Global AIDS Coordinator U.S. State Department Washington DC USA

**Keywords:** differentiated service delivery, HIV treatment, National TB and Leprosy Programme, patient‐centred care, persons living with HIV, tuberculosis, tuberculosis treatment

## Abstract

**Introduction:**

Providing more convenient and patient‐centred options for service delivery is a priority within global HIV programmes. These efforts improve patient satisfaction and retention and free up time for providers to focus on new HIV diagnoses or severe illness. Recently, the coronavirus disease 2019 (COVID‐19) pandemic precipitated expanded eligibility criteria for these differentiated service delivery (DSD) models to decongest clinics and protect patients and healthcare workers. This has resulted in dramatic scale‐up of DSD for antiretroviral therapy, cotrimoxazole and tuberculosis (TB) preventive treatment. While TB treatment among people living with HIV (PLHIV) has traditionally involved frequent, facility‐based management, TB treatment can also be adapted within DSD models. Such adaptations could include electronic tools to ensure appropriate clinical management, treatment support, adherence counselling and adverse event (AE) monitoring. In this commentary, we outline considerations for DSD of TB treatment among PLHIV, building on best practices from global DSD model implementation for HIV service delivery.

**Discussion:**

In operationalizing TB treatment in DSD models, we consider the following: *what* activity is being done, *when* or how often it takes place, *where* it takes place, *by whom* and *for whom*. We discuss considerations for various programme elements including TB screening and diagnosis; medication dispensing; patient education, counselling and support; clinical management and monitoring; and reporting and recording. General approaches include multi‐month dispensing for TB medications during intensive and continuation phases of treatment and standardized virtual adherence and AE monitoring. Lastly, we provide operational examples of TB treatment delivery through DSD models, including a conceptual model and an early implementation experience from Zambia.

**Conclusions:**

COVID‐19 has catalysed the rapid expansion of differentiated patient‐centred service delivery for PLHIV. Expanding DSD models to include TB treatment can capitalize on existing platforms, while providing high‐quality, routine treatment, follow‐up and patient education and empowerment.

## 1 INTRODUCTION

Before severe acute respiratory syndrome coronavirus 2 (SARS‐CoV‐2), the virus that causes coronavirus disease 2019 (COVID‐19), tuberculosis (TB) was the world's deadliest infectious disease; TB remains the leading cause of death for people living with HIV (PLHIV) [1, 2]. In 2019, an estimated 208,000 PLHIV died of TB globally. Only 71% of the estimated individuals with incident TB were treated, and treatment success only reached 56% among PLHIV [1]. Approximately 1.4 million fewer TB cases were reported globally in 2020 partly because COVID‐19 reduced access to health facilities and triggered commodity stock‐outs [3, 4, 5]. While more data are needed to characterize and quantify the impact of COVID‐19 on TB diagnosis, treatment and prevention, modelling studies have suggested that the number of people developing TB could increase by more than 1 million per year between 2020 and 2025 [1, 6].

In HIV care, differentiated service delivery (DSD) includes tailored adaptations to meet the needs and preferences of PLHIV, while also streamlining care in the context of limited human resources and infrastructure [7]. DSD models are increasingly being adopted, including recent scale‐up of multi‐month dispensing (MMD) options for antiretroviral therapy (ART) and TB preventive treatment (TPT) for patients supported through the US President's Emergency Plan for AIDS Relief [8, 9, 10, 11]. To date, most DSD models have primarily served PLHIV considered “stable on ART” by reducing the frequency of clinic visits and ART dispensation (e.g. every 3–6 months) or by making services available in communities. DSD models improve patient retention and satisfaction, reduce patient costs (e.g. transportation or lost labour) and free up space and time in facilities for health providers to focus on PLHIV with new diagnoses or who require intensive care [7, 9]. However, PLHIV with a TB disease diagnosis are often ineligible for these models because they are not considered “stable on ART”, which necessitates biweekly or monthly visits to a health facility for close management and follow‐up while they receive TB treatment [12, 13, 14]. To help patients with TB access care, the World Health Organization's Global TB Programme has prioritized patient‐centred care, and directly observed treatment for TB has moved from strictly facility‐based to community‐based or remote/virtual models, though frequent and close interaction has remained a hallmark of this approach [14, 15].

This paradigm is now changing in the context of COVID‐19, which has pushed programmes to decrease patient contact with health facilities to reduce the risk of COVID‐19 transmission for both patients and providers [8, 9]. In HIV programmes, eligibility criteria have been expanded across many countries to ensure that all patients have a continuous supply of critical medications despite COVID‐19 disruptions and lockdowns [8, 9]. These policy changes have resulted in dramatic scale‐up of MMD options for ART, cotrimoxazole and TPT paired with direct delivery to patient communities or homes and greater reliance on virtual treatment support for adherence and adverse event (AE) monitoring [8, 16].

TB treatment delivery could also be adapted to this new environment to mitigate disruptions to patients’ treatment courses and to support long‐term gains in TB epidemic control [8, 17, 18, 19, 20]. Underscored by new Joint United Nations Programme on HIV/AIDS targets for 2025, the vision of DSD can promote sustainable patient‐centred care by integrating treatment services for HIV and other diseases, such as TB [21, 22]. Drawing on principles of HIV DSD, we propose that differentiated TB treatment for PLHIV – while ensuring appropriate TB clinical care, treatment support and AE monitoring – could be implemented and sustainably scaled and maintained after the COVID‐19 pandemic. Although we propose scaling up TB treatment within HIV DSD models, this approach could also improve treatment outcomes for HIV‐negative persons with TB, who comprise >90% of global TB cases. Similarly, while we focus on treatment of drug‐sensitive TB, many of the principles described could be applied in all‐oral treatment of drug‐resistant TB. We describe overarching considerations for TB treatment delivery within DSD models, a conceptual example among PLHIV and an early implementation experience in Zambia among people treated for TB irrespective of HIV status.

## 2 DISCUSSION

### 2.1 General principles and considerations for incorporating TB treatment into differentiated HIV service delivery models

#### 2.1.1 DSD framework for TB treatment and alignment with HIV care

Important considerations for operationalizing TB treatment in DSD models include the following: *what* activity is being done, *when* or how often it takes place, *where* it takes place, *by whom* and *for whom* [23]. We propose the initial step in incorporating TB treatment into DSD models for PLHIV is to assess the policy and structure of current DSD models to determine how these could be leveraged and/or adapted. For PLHIV already established in a DSD model before receiving a TB diagnosis, minimizing changes to their chosen model by aligning timing and location of TB service delivery with HIV service delivery is integral to preserving the intent of DSD enrolment. Consultation with patients and civil society is critical to ensure that the patient‐centred nature of a given model is optimized and adapted as needed.

#### 2.1.2 TB screening and diagnosis

Because expanding DSD models for PLHIV may mean less frequent facility‐based interactions, routine high‐quality screening for TB disease can be performed in other settings and/or virtually (e.g. through virtual platforms or mobile technology such as texting or telephone check‐ins) [24]. Given their common symptomatology, TB symptom screening and evaluation could be coupled with COVID‐19 screening and testing in or outside health facilities. TB symptom screening can be provided for PLHIV during standardized virtual follow‐ups, community drug distribution or by patients themselves or treatment supporters (e.g. peer educators or community health workers). Confirmatory TB diagnostic testing, in contrast, is complicated, and should still be performed by a designated health provider in accordance with national guidelines. However, sputum specimens, samples for lateral flow urine lipoarabinomannan assays and digital chest X‐rays could be collected in community settings to increase patient convenience by leveraging networks of treatment supporters and existing referral and transport systems (e.g. for HIV viral load or COVID‐19 testing). National TB programmes have long used treatment supporters, including for sputum collection, and these innovations can be incorporated into new DSD models. Patient preferences of DSD modality, treatment supporter training, timely sample collection and referral of results for treatment evaluation (e.g. through point‐of‐care or reliable digital technologies) are important considerations.

### 2.2 TB treatment initiation

#### 2.2.1 Medication dispensing

A key consideration in adapting TB treatment delivery is determining how many doses of TB medication will be dispensed at treatment initiation. If drug supply permits, longer TB medication dispensing intervals – even if clinical disease severity necessitates more frequent clinical encounters – is the best practice to ensure uninterrupted treatment (“decoupling” refill frequency from clinical assessment frequency). One strategy could be to provide 2 months of TB medication at initiation to last through the full intensive treatment phase. Dispensation could occur at health facilities, community pharmacies, other community distribution points or in home‐based settings (e.g. visit by supporter or mail delivery). Aligning TB medication and ART dispensing location and timing, including options for expanded pick‐up hours or fast‐tracked services (i.e. services for which patients do not need to see a clinician or provider to access) will substantially improve patient outcomes [25].

### 2.3 Patient education and counselling at TB treatment initiation

With less frequent facility‐based interactions between health providers and patients, collaborative discussions about what to expect in treatment, especially a focus on empowering patients to commit to treatment completion, are critically important at treatment initiation and throughout the treatment course [26, 27, 28, 29]. Standardized and comprehensive education and counselling include emphasizing the importance of adherence to TB treatment and potential complications and consequences of missed doses or discontinuation. It is critically important that patients receive counselling and informational materials for home review on TB treatment related AEs, with clear instructions to contact a designated treatment supporter or health provider at the onset of any worrisome sign or symptom. Patient education should also include discussion of symptoms of immune reconstitution inflammatory syndrome, especially for those who are newly initiating ART. While provision of standardized counselling and education is not novel, it is not standard practice in many TB treatment programmes, but should be ensured.

### 2.4 TB treatment management

#### 2.4.1 Medication dispensing

Timing, frequency and location of TB medication dispensing can be adapted to local context and patient needs and align with ART dispensing location and frequency. For example, after completing the intensive treatment phase with demonstrated response to treatment, a patient could return to the health facility and receive 4 months of TB medication to cover the entire continuation phase, with subsequent check‐ins delivered in the community or virtually. If more frequent refill dispensation is needed, this could occur at alternative, more convenient locations and/or be performed by a designated alternate provider (e.g. pharmacist or treatment supporter).

#### 2.4.2 Monitoring treatment response and effectiveness

Despite reductions in facility‐based encounters, changes in a patient's symptoms while on TB treatment can be monitored through frequent, standardized and virtual check‐ins [30]. As with diagnosis, specimen collection to monitor bacteriologic response can be performed in the community setting. A 2‐month follow‐up clinic visit and visit at the end of treatment with a health provider would enable bacteriologic testing and physical examination to assess response to TB treatment.

In cases of TB treatment non‐response, it is important to identify any underlying barriers to adherence or risk factors for drug resistance. The provider could then adapt the patient's management plan with more frequent in‐person or virtual monitoring, additional diagnostic evaluation for drug resistance, a modified treatment regimen and/or adherence counselling and additional support.

#### 2.4.3 Monitoring adherence and AEs

For ongoing adherence counselling and AE monitoring, patients could be linked to a designated treatment supporter, existing support group, virtual support or some combination. [25, 31, 32]. Several modalities for virtual monitoring and support for TB patients have been shown to provide higher patient and provider satisfaction, cost savings and high rates of treatment adherence and completion [33, 34, 35]. Digital adherence technologies such as pill sleeves or boxes that provide a proxy for medication use may be another feasible and acceptable alternative [36]. The frequency of check‐ins could vary depending on the patient's clinical status and preference as well as method of interaction (e.g. daily text messaging adherence reminders and AE screens could be paired with monthly video or phone discussions with a health provider).

### 2.5 Recording and reporting

Characteristics of successful TB treatment programmes include appropriate and timely data collection, dissemination and use for continuous programme improvement. Programmes may need to create or adapt fields in patient charts, aggregate registers and electronic medical records to ensure capture of TB treatment model, drug dispensation, adherence and AE monitoring and treatment outcomes. Digital technologies and platforms can enable automated screening and adherence questionnaires to enhance reporting completeness; such platforms should be considered for patients with access to mobile phones [36]. As new options for TB treatment delivery are introduced, routinely assessing patient outcomes within each DSD model ensures model non‐inferiority, patient satisfaction and continuous programme quality improvement.

### 2.6 Examples of TB treatment integration into DSD models

#### 2.6.1 Conceptual model incorporating TB treatment into 3‐month ART MMD

**Figure 1:**
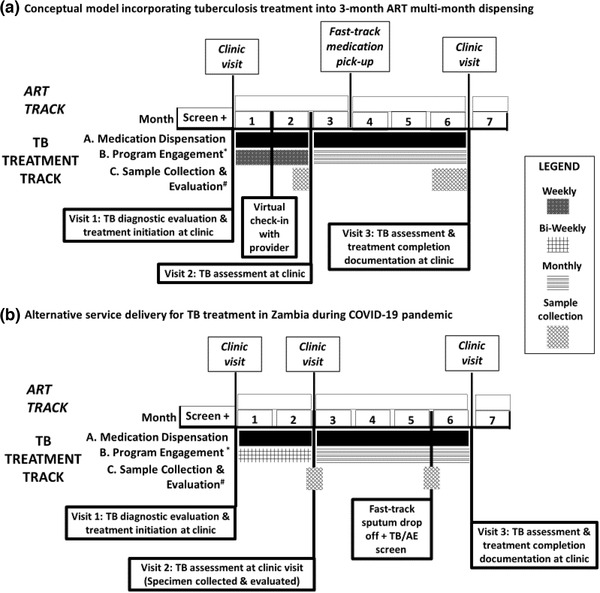
Examples of tuberculosis (TB) treatment integration in differentiated service delivery models; (A) This diagram defines the elements of the model and their timing/frequency. There are flexibilities inherent in this model with regard to who will carry out each task and in what setting. For example, medication dispensation could occur at the clinic, pharmacy or at community distribution points. Likewise, programme engagement (check‐ins, adherence support) could be virtual, community‐based or a combination of these. Sample collection and evaluation will most often be based in the clinic setting, but there may be models or specific situations that entail community‐based specimen collection. For example, ^*^Programme engagement consists of virtual or community contact by a treatment supporter to monitor for adherence and adverse events. ^#^Sample is collected in the community and prior to clinic visit for a TB assessment. Fast track consists of clinic visits every 6 months with fast‐track medication pick‐up at 3 months in between clinic visits. HIV treatment: 6‐month clinical visit per year and clinical services/medication refills are fast tracked (expedited) at 3 months. TB treatment: consists of three in‐person clinic visits and one virtual check‐in with a health provider. TB medication is dispensed twice, for 2 and 4 months. Sample collection and evaluation is performed prior to the TB assessment clinic visits. Programme engagement is conducted by a treatment supporter weekly during the intensive phase and monthly during the continuation phase. (B) HIV treatment: PLHIV receiving TB treatment through this model have their HIV medication aligned to the TB treatment arm for this 6‐month period (2 months/4 months) then return to 6 months of dispensation following treatment completion. TB treatment: At treatment initiation, patients receive 2 months of intensive phase treatment (rifampin, isoniazid, pyrazinamide, ethambutol) and receive bi‐weekly virtual check‐ins. At month 2, patients return to clinic for evaluation and switch to continuation treatment (rifampin, isoniazid); if they are smear‐negative and responding well to treatment, they receive 4 months of medication and virtual check‐ins revert to monthly. If a patient has not converted to smear‐negative status at month 2 or revert positive at month 5, they are evaluated for drug resistance and returned to TB treatment standard of care based in the clinic. AE, adverse event; ART, antiretroviral therapy; HIV, human immunodeficiency virus; PLHIV, persons living with HIV; TB, tuberculosis.

A conceptual model for incorporating TB treatment into standard MMD for ART is provided in the Figure 1A. After receiving a TB diagnosis, the patient is seen at the clinic and prescribed 1 or 2 months of intensive phase TB medications. With intensive counselling on adherence and potential AEs by the provider, the patient is linked to a treatment supporter. During the 2‐month intensive phase, the patient participates in weekly community‐based or virtual AE screening and adherence counselling with a treatment supporter and a 1‐month virtual check‐in by the health provider. Before the 2‐month clinic visit, sputum specimen collection occurs in the community for evaluation by smear microscopy and/or culture, in time for provider assessment and discussion of treatment effectiveness at that visit [37]. If the treatment is effective, the patient receives a 4‐month supply of TB continuation phase treatment. At the patient's final TB clinic visit, the patient is evaluated for TB treatment completion and success by a provider (again requiring specimen collection before this visit). Automated adherence reminders via texting and virtual check‐ins via phone calls occur at least weekly during the intensive phase and at least monthly throughout the continuation phase. These modalities could be strengthened with backup contact plans, either through contact with treatment supporters or through household visits [25, 38]. Provision of dedicated mobile phones and airtime to clinic staff ensures consistent virtual follow‐up. A variation in this model to further align TB and HIV treatment dispensation could be to dispense 4 months of TB treatment medication and ART at the 2‐month clinic visit. It is important to document all encounters, especially community‐based and virtual clinical encounters via paper‐based or electronic systems. Throughout, the patient is engaged as a partner to maintain patient activation and self‐efficacy, to maximize treatment success even as in‐person clinical interactions may be limited.

#### 2.6.2 Early Implementation in Zambia

Within 3 months of implementation of COVID‐19 mitigation measures in Zambia, TB diagnosis fell by 30% while TB treatment loss to follow‐up increased slightly compared to previous years. In the context of COVID‐19, Zambia modified delivery of TB treatment to address these challenges [39]. As shown in Figure 1B, updated guidance released in April 2020 allows MMD for drug‐susceptible TB treatment (2 months at initiation followed by 4 months after clinical follow‐up), fast‐track sputum specimen drop‐off at the facility (at month 5) and virtual (texting/phone) bi‐monthly and monthly check‐ins for symptoms and AEs during intensive and continuation phases, respectively. The guidance emphasizes selecting and educating a home‐based treatment supporter to provide daily adherence support; however, the guidance does not explicitly mandate alignment of TB care with ART service delivery.

Several virtual discussions held with health staff across Zambia on implementation of new guidelines allowed for real‐time troubleshooting. The National TB and Leprosy Program (NTLP) hosted a weekly TB Situation Room to monitor and mitigate the impact of COVID‐19 on case‐finding and other TB services. Using this virtual platform, they addressed commodity distribution issues and granted facilities maximum flexibility in implementing DSD models. Limited direct training was provided to the health providers and data clerks, which resulted in data irregularities and fewer virtual check‐ins than planned. Virtual monitoring was complicated by lack of dedicated phones and airtime for TB departments and difficulty reaching patients. Since then, virtual trainings have been scaled up, and revised guidance has been developed to address these issues.

Since April 2020, Zambia's NTLP estimates that 80% of all health facilities providing TB services provide TB treatment through a DSD model, with nearly all their patients enrolled. Common model variations include 1‐month drug dispensation during the intensive treatment phase and 2‐month dispensation during the continuation phase because of stock limitations. Data from 10 early‐adopter sites in Lusaka Province presented at a TB Situation Room meeting showed that all 1449 patients starting TB treatment between 1 March and 31 May 2020, were enrolled in some form of DSD model for TB treatment [39], and that all patients received at least one treatment support visit virtually or in the community. Among enrolled patients, 1396 (96.3%) had a documented treatment outcome, but 53 (3.7%) were lost to follow‐up. Among those with a documented outcome, 1334 (95.6%) completed treatment, 56 (4.0%) died, 5 (0.4%) were diagnosed with drug‐resistant TB and 1 (0.1%) discontinued treatment due to a severe AE.

After implementing this DSD model alongside the NTLP TB Situation Room which managed site‐specific issues and challenges, TB case notification and treatment success rates have rebounded to level or surpass pre‐COVID performance. Although these guideline changes were an emergency measure in response to the COVID‐19 pandemic, the NTLP is planning more rigorous programme evaluations to assess patient outcomes and the impact and long‐term utility of these adaptations.

## 3 CONCLUSIONS

COVID‐19 has catalysed rapid global scale‐up of differentiated, patient‐centred service delivery for PLHIV. Expanding DSD models to include TB treatment can capitalize on existing platforms while providing high‐quality, routine treatment follow‐up and patient education and empowerment. These efforts can be supported by existing networks of treatment supporters, systems for sample collection and transport and mobile technologies. Adapting the existing provider–patient interface for TB treatment could not only increase patient convenience and satisfaction but also reduce patient and health system costs and other barriers to adherence. Incorporating these options into health programmes would contribute to realizing the goal of integrated, patient‐centred, sustainable care.

## COMPETING INTERESTS

The authors declare no conflicts of interest.

## AUTHORS' CONTRIBUTIONS

CHT, BKM, IP and AT. Boyd conceived and wrote the first draft of the manuscript. PL, SS, IO, TA‐S, SAM and AD reviewed and edited the final version of the manuscript.
